# Laboratory-Scale Isolation of Insect Antifreeze Protein for Cryobiology

**DOI:** 10.3390/biom9050180

**Published:** 2019-05-09

**Authors:** Heather E. Tomalty, Laurie A. Graham, Robert Eves, Audrey K. Gruneberg, Peter L. Davies

**Affiliations:** Department of Biomedical and Molecular Sciences, Queen’s University, Kingston, ON K7L 3N6, Canada; 2het@queensu.ca (H.E.T.); grahamla@queensu.ca (L.A.G.); evesr@queensu.ca (R.E.); 15akg3@queensu.ca (A.K.G.)

**Keywords:** antifreeze protein, ice-affinity purification, thermal hysteresis, cryopreservation

## Abstract

Micromolar concentrations of hyperactive antifreeze proteins (AFPs) from insects can prevent aqueous solutions from freezing down to at least −6 °C. To explore cryopreservation of cells, tissues and organs at these temperatures without ice formation, we have developed a protocol to reliably produce ultrapure *Tenebrio molitor* AFP from cold-acclimated beetle larvae reared in the laboratory. The AFP was prepared from crude larval homogenates through five cycles of rotary ice-affinity purification, which can be completed in one day. Recovery of the AFP at each step was >90% and no impurities were detected in the final product. The AFP is a mixture of isoforms that are more active in combination than any one single component. Toxicity testing of the purified AFP in cell culture showed no inhibition of cell growth. The production process can easily be scaled up to industrial levels, and the AFP used in cryobiology applications was recovered for reuse in good yield and with full activity.

## 1. Introduction

Antifreeze proteins (AFPs) were originally discovered in Antarctic marine fishes as a mechanism by which the fish were prevented from freezing in icy seawater [1]. The AFPs bind at intervals over the surface of seed ice crystals and retard ice growth by the Gibbs–Thompson effect [2]. This adsorption–inhibition effect causes a depression of the solution freezing point below the ice melting point, which is termed thermal hysteresis (TH), and led to the suggestion that AFPs could be used to extend freeze protection to other fishes [3]. Overwintering terrestrial insects are typically challenged by much lower temperatures than the −2 °C of icy seawater and have evolved more potent AFPs [4]. The ability to lower freezing points below those attained with fish AFPs at lower protein concentrations led to the insect AFPs being termed ‘hyperactive’ [5]. The basis for this hyperactivity seems to correlate with the ability of the AFP to bind to the basal plane of ice [6,7]. However, it should be stressed that not every AFP that binds the basal plane is hyperactive [8]. The recent characterization of a variety of hyperactive AFPs in insects [9] has stimulated ideas for their applications in cryobiology [10,11,12]. 

We are currently exploring the use of AFPs to depress the freezing point of cells, tissues and organs below −6 °C without the formation of ice. Although, production of large amounts of hyperactive AFPs may ultimately be done by industrial fermentation, previous attempts at recombinant expression were labor-intensive and time-consuming due to the highly disulfide- bonded structure of the AFPs [13,14]. We have developed an intermediate process at a laboratory scale that can produce enough natural AFPs for ‘proof-of principle’ cryobiology experiments. Our process avoids the need to refold the AFPs and takes advantage of the ease of rearing the domesticated yellow mealworm beetle, *Tenebrio molitor*, on inexpensive bran and water. Both the developmental stage and duration of cold acclimation needed to optimize AFP production in beetle larvae were previously determined [15], and larvae at peak AFP production can be frozen and stockpiled. 

*T. molitor* AFP (*Tm*AFP) is a mixture of similar isoforms in the 7–9 kDa mass range, some of which are glycosylated [16]. It has been reported that the mixture of homologous AFP isoforms in the closely related beetle, *Dendroides canadensis*, has a synergistic effect on TH activity [17]. To capitalize on this effect and to achieve isolation of all isoforms at the same time, we have used ice-affinity purification (IAP) [18], but with the recent modification of doing this in a spinning round-bottom flask to increase the surface area over which the AFP isoforms are extracted into ice [19]. 

Here we document the purity and yield of the natural AFP produced without chromatography using an inexpensive apparatus. We report that *Tm*AFP produced by our process has no obvious toxicity with tissue culture cells when used at levels that can depress the freezing point of the cell medium to below −6 °C. We discuss the options for scaling this process to industrial levels. 

## 2. Materials and Methods 

### 2.1. Beetle Cultivation 

The *T. molitor* colony was maintained on wheat bran that was previously stored at −20 °C for at least a week to ensure that stored-grain pests were killed. The bran was sieved using a riddle with 3/32″ (2.4 mm) mesh and ~3 L was added to 9 L rectangular plastic storage bins without lids. Water was provided by wetting paper towels, which overlaid the bran, three times a week as previously described [15]. A new production cycle was started each week by adding ~100 adults. These beetles were removed after one week of egg laying, to maximize synchronous development of the larvae. Once some larvae began to pupate (after 15 to 19 weeks), the bran was sieved as above to isolate the larvae, many of which would be in their final instar. They were added to a fresh bin of bran without provision of water (to minimize the growth of fungus), and were placed at 4 °C for 4 weeks to enhance the production of AFP. Finally, the larvae were again harvested by sieving, and were stored frozen at −80 °C. 

### 2.2. Tenebrio Molitor Antifreeze Protein (TmAFP) Extraction

Frozen mealworm larvae (100 g) were homogenized for 30 s using a standard kitchen blender on high setting in 300 mL lysis buffer (50 mM Tris-HCl (pH 7.8), 100 mM NaCl, 1 mM phenylthiocarbamide, 1 mM ethylenediaminetetraacetic acid (EDTA), 0.1 mM phenylmethylsulfonyl fluoride (PMSF)) pre-chilled to 4 °C. Phenylthiocarbamide was added to inhibit phenoloxidases, and PMSF (added just prior to use) plus EDTA were used to inhibit serine- and metallo-proteinases, respectively. The larval homogenate was centrifuged at 25,000× *g* for 30 min at 4 °C. The surface lipid layer was skimmed off from the centrifuge bottles and residual lipid was removed by filtration through glass wool into a cooled beaker. The supernatant volume (typically ~250 mL) was made up to 400 mL with deionized, filtered water and kept on ice prior to ice-affinity purification. 

### 2.3. Rotary Ice-Affinity Purification

Ice shells were prepared in 1-L round-bottom flasks by adding cold, deionized, filtered water (200 mL) into the flask while spinning it in a −80 °C ethanol bath in a Styrofoam bucket for 50–80 s. The excess water was poured off into a measuring cylinder to calculate by difference the volume of the ice shell, which should be 30–50 mL. The flask was then spun in the ethanol bath for another 30 s or more while the ice shell hardened as evidenced by cracking of the ice.

Ice-cold, diluted supernatant (200 mL) was added to the flask containing the ice shell. Two flasks were used for each 100 g of insects and these were rotated at ~60 rpm in separate cooling baths set at −1.6 °C. After ~1.25 h the liquid fraction in each had typically been reduced to ~100 mL, with an equal volume incorporated into the ice. Bath temperature and extraction duration can be slightly adjusted as needed to achieve ~50% incorporation of the liquid fraction. The liquid fraction was decanted from the flask into a measuring cylinder to calculate by difference the volume of supernatant incorporated into the ice shell. 

Each ice shell was melted and the volume made up to 200 mL by adding 10 mL of a 20X stock of melting buffer (0.5 M Tris-HCl (pH = 7.8), 1 M NaCl, 10 mM phenylthiocarbamide, and 10 mM EDTA) along with cold, deionized, filtered water to achieve a final solute concentration of 25 mM Tris-HCl (pH = 7.8), 50 mM NaCl, 0.5 mM phenylthiocarbamide, and 0.5 mM EDTA. *Tm*AFP was re-extracted into a second ice shell at −1.6 °C for ~ 1 h while impurities were excluded into the liquid fraction. This process was repeated a third time in exactly the same manner as the second extraction. Thereafter, a fourth extraction was performed, but with the omission of phenylthiocarbamide from the melting buffer. For the fifth and final extraction, only NH_4_HCO_3_ was added, to 20 mM, allowing the option to lyophilize the *Tm*AFP while removing this volatile salt. 

### 2.4. Concentration of Final Ice Fraction

Final ice fractions were melted, combined and brought to 20 mM NH_4_HCO_3_ using 1/25th volume of a 500 mM stock. They were concentrated to ~50 mL at 4 °C using a 500-mL Amicon Stirred Cell (Millipore Sigma, Canada) and an ultrafiltration membrane disc (3,000 MWCO). Samples were further concentrated using Amicon centrifugal filters (Millipore Sigma, Canada; 3,000 MWCO) and flushed twice with 15 mL of 20 mM NH_4_HCO_3_ to remove any trace low-molecular-weight impurities. Samples were then centrifuged for 1.5 h at 14,500× *g* at 4 °C to pellet any insoluble debris. Concentrated samples were flash frozen and stored at −80 °C, with aliquots sent for amino acid analysis to determine final AFP concentration. 

### 2.5. Thermal Hysteresis (TH)

TH activity was measured using a nanolitre osmometer [20]. Once an accurate concentration of a purified *Tm*AFP stock solution was obtained by amino acid analysis, a plot of TH vs. protein concentration was prepared. From this it was possible to interpolate the *Tm*AFP concentration of an unknown solution from the TH activity alone.

### 2.6. Amino Acid Analysis

Amino acid analysis was performed at the SPARC Biocentre (Sick Kids, Toronto ON). Samples were vacuum dried, suspended in 6N HCl containing 1% phenol, and hydrolyzed for 24 h at 110 °C under nitrogen. Following hydrolysis, samples were vacuum dried to remove excess HCl, resuspended in a re-drying solution of methanol: water: trimethylamine (2:2:1) and dried under vacuum for 15 min. Samples were derivatized for 20 min at room temperature in methanol: water: trimethylamine: phenylisothiocyanate (7:1:1:1). The derivatizing solution was removed under vacuum, and the samples were washed with re-drying solution and vacuum dried for an additional 15 min. Samples were then dissolved in diluent and injected into an ethylene bridged hybrid C18 column to run on a modified Pico-Tag gradient at 48 °C, with phenylthiohydantoin (PTH)-derivatized amino acid detection occurring at 254 nm. A Waters Acquity Ultra-High Performance Liquid Chromatography (UPLC) System was used to control the chromatography. Cysteine quantification for select samples was performed by performic acid oxidation prior to hydrolysis.

### 2.7. Toxicity Assays in HEK 293 T Cell Line

HEK 293 T cells, obtained from Dr. Peter A. Greer (Queen’s University), were grown to 70–80% confluence in Dulbecco’s modified Eagle’s medium (DMEM) supplemented with 10% fetal bovine serum (FBS) under standard growth conditions (37 °C, 5% CO_2_). Cells were harvested and 96-well plates seeded at initial densities of 1 × 10^4^ to 5 × 10^4^ cells/well. Regular growth medium (DMEM + 10% FBS) was added to control wells, while experimental wells contained either 50 µg/mL or 100 µg/mL *Tm*AFP suspended in DMEM + 10% FBS. Plates were incubated at 37 °C, 5% CO_2_ and MTT assays [21] were performed on individual plates following 24, 48, and 72 h of growth. Briefly, cell medium was removed from each well and phenol red-free DMEM with 0.5 mg/mL MTT was added. Plates were incubated at 37 °C for 3 h, after which the MTT-supplemented medium was removed and DMSO added. Plates were shaken for 1 h at room temperature to solubilize the formazan. Absorbance readings on 100 µL samples were taken at 570 and 690 nm and % cell viability calculated.

### 2.8. Recycling of Used TmAFP

Spent culture medium from cell incubations designed to test *Tm*AFP for toxic effects were frozen. Once a sufficient volume was accumulated, it was thawed, pooled and AFP was recovered by four-rounds of IAP as described for the extraction from the beetle larvae.

## 3. Results

### 3.1. Maintaining the Yield of Larvae

**Figure 1:**
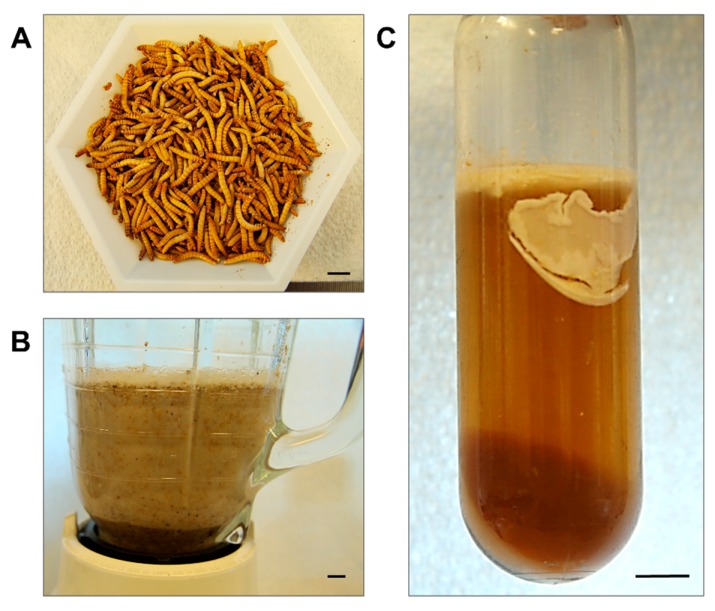
Preparation of *T. molitor* larval extract. (**A**) Cold-acclimated larvae (100 g). (**B**) Larval homogenate after blending for 30 s in 300 mL of lysis buffer. (**C**) Homogenate after centrifugation for 30 min. Scale bars in the right corner of each photograph represent 1 cm.

An observation we made from keeping the beetle colony in the lab for decades is the tendency to select for early pupation. The weight at which most larvae (Figure 1A) entered pupation steadily decreased from 120–200 mg to 80–120 mg over the years, because we had been using the first beetles to emerge from pupation to repopulate the colony. While this shortened the generation time, it decreased the average weight of the larvae from which we were extracting AFP. Therefore, we reversed this trend by selecting the larger late-instar larvae to become the breeding population. Within three generations, the majority of the larvae did not pupate prior to reaching 120 mg, so this simple procedure increased the yield of starting material for the AFP extraction.

Centrifugation of the blended larvae (Figure 1B) with average weights of 115 mg produced a light brown supernatant that was easily decanted from the darker pellet and straw-coloured lipid layer that adhered to side of the centrifuge tube near the top of the liquid (Figure 1C).

### 3.2. Ice-Affinity as the Sole Purification Procedure

**Figure 2:**
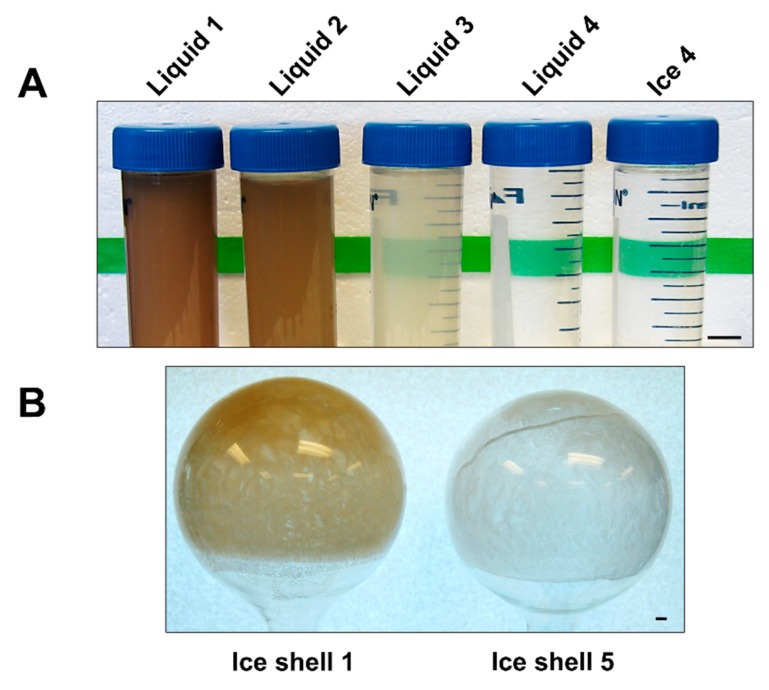
Serial ice-affinity purification of *T. molitor* antifreeze protein (*Tm*AFP). (**A**) Liquid fractions showing the removal of contaminants through the first four rounds of ice-shell affinity purification. (**B**) Ice shell #1 compared to ice shell #5. Scale bars in the right corner of each photograph represent 1 cm.

The principle behind ice-affinity purification is that AFPs bind to ice and are overgrown, while impurities are excluded into the liquid above the ice [18,19]. From our experience, the partitioning of AFPs into ice during rotary affinity purification can be quite variable from one AFP type to another. We had previously observed *Tm*AFP was very efficiently incorporated into the ice shell, whereas in comparison only 50% of fish type III AFP was incorporated [19]. For this reason we elected to use rotary IAP as the sole purification method for *Tm*AFP and repeat the extractions until impurities were no longer detectable in the ice fraction. Visual inspection of the excluded liquid fractions showed a dramatic reduction in the colour and opacity of the liquid fraction by the third extraction (Figure 2A). Impurities were not visible by eye in liquid fraction 4, whereas at the previous stage, liquid fraction 3 still had noticeable discolouration. With each extraction, the ice shells also became cleaner. The first ice shell had a noticeable brown tinge, but the last two ice shells were colourless and indistinguishable by appearance from pure water when thawed (Figure 2B). 

**Figure 3:**
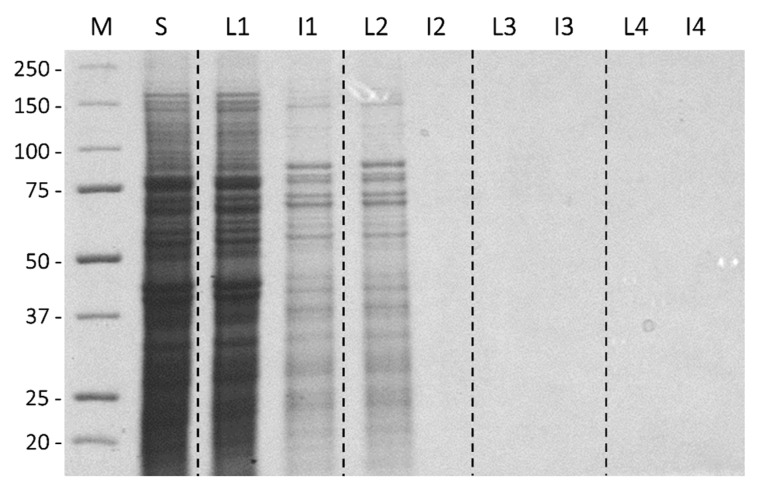
Coomassie blue-stained SDS-PAGE analysis of serial ice-affinity purifications. Lanes were loaded with liquid (L) and ice (I) fractions on an equal volume basis (lanes 3–10) relative to the starting material (S) (lane 2). Samples represented in each lane are as follows: protein markers in kDa (M), supernatant (S), liquid 1 (L1), ice 1 (I1), liquid 2 (L2), ice 2 (I2), liquid 3 (L3), ice 3 (I3), liquid 4 (L4), ice 4 (I4). Vertical dotted lines partition the lanes into sets of liquid and ice fractions from each ice affinity purification step.

Protein assays by the Bradford method supported these visual observations (Table 1). The starting extract contained 44,300 mg of protein, 93% of which was excluded into liquid fraction 1. Thereafter, 5% of the total was found in liquid fraction 2; 1% in liquid fraction 3; and 0.1% into liquid fraction 4. Liquid fraction 5 had no detectable protein present. The last ice fraction to have detectable amounts of protein present was ice fraction 3 with 34 mg. Note, because of *Tm*AFP’s atypical amino acid composition, it does not bind Coomassie blue and it was not possible to assay the AFP using the Bradford reagent or to see it on sodium dodecyl sulfate polyacrylamide gel electrophoresis (SDS-PAGE) stained with this dye [13,14]. These methods were only used here to track the removal of impurities. When these fractions were analysed by SDS-PAGE and Coomassie blue staining, the supernatant fraction from the larval homogenate had a vast number of different proteins ranging up to ~175 kDa (Figure 3, lane 1). The first liquid fraction (lane 2) reflected this complexity and was quantitatively almost indistinguishable in band intensity due to 90% of the total protein being excluded into this fraction. In contrast, the first ice fraction (lane 3) had only a tenth as much protein. This pattern was repeated through another extraction where the second liquid (lane 4) fraction was almost indistinguishable from the first ice fraction (lane 3), and the second ice fraction (lane 5) had barely detectable amounts of protein. Because *Tm*AFP is not stained by Coomassie blue, its recovery was assessed by TH activity. Ice fractions consistently had ~3.5 °C of TH activity (Table 1). In comparison, the first two liquid fractions had only 0.24 °C and 0.16 °C of TH, respectively, representing 2.4% and 1.6% of the AFP as determined from a standard curve of TH (see below). These amounts are not worth recovering by back-extraction. Subsequent liquid fractions had negligible TH values of <0.1 °C. 

### 3.3. Phenoloxidase Inhibition 

Phenoloxidase inhibition is a critical part of the purification. Insects have a well-developed clotting mechanism based on melanization to seal wound sites and prevent bacterial infections [22]. In response to injury, phenoloxidases are generated from proprotein precursors and catalyse the polymerization of phenolic compounds to form dark-coloured insoluble melanin. The addition of 1 mM phenylthiocarbamide (also called phenylthiourea) was effective in preventing melanization. However, in some of the initial *Tm*AFP preparations, this inhibitor was omitted after the second ice shell was formed, with the consequence that a trace amount of melanin was detected in the subsequent fractions, as for example in liquid fraction 3 in Figure 2A. Using the inhibitor in the third round of ice-affinity extraction eliminated this problem. However, we also observed that trace amounts of phenylthiocarbamide were inhibitory to the HEK 293T cells in culture (data not shown). For this reason, phenylthiocarbamide was omitted from the fourth and fifth ice shell extractions.

### 3.4. TmAFP Recovery and Yields

As mentioned earlier, because of its unusual amino acid composition biased towards short-chain amino acids, where Thr, Cys, Asx, Ala, Gly and Ser make up 80% of the total, and aromatic and basic residues are underrepresented (Table 2), it was not possible to track the recovery and purification of *Tm*AFP by SDS-PAGE and Coomassie-blue staining or by conventional protein detection assays. Amino acid analysis was, however, a good indicator of purification because of *Tm*AFP’s distinct amino acid composition. Typical proteins are rich in leucine and isoleucine, but these residues are minimally present (each < 1%) in the purified *Tm*AFP, indicating that there was little or no contamination by other proteins. Also, the compositions of all *Tm*AFP preparations were remarkably consistent from one batch to another, as shown by the four examples in Table 2. These compositions were compared to predicted values calculated from the relative proportions (50:25:15:10) of the four most abundant *Tm*AFP isoforms (4-9, D-16, 1-2 and A-3, respectively) isolated from larval hemolymph [16]. In the four experimental analyses, the percentage of alanine ranged from 10.1 to 10.7% in comparison to a predicted value of 11%. Threonine ranged from 20.9 to 22.2% when the predicted value was 21.9%. For one of these analyses (4B) the cysteine content was measured after oxidation to cysteic acid. Based on amino acid composition the average yield of *Tm*AFP from 100 g of larvae was 4.6 mg, with a range of 3.2 to 5.9 mg. 

**Figure 4:**
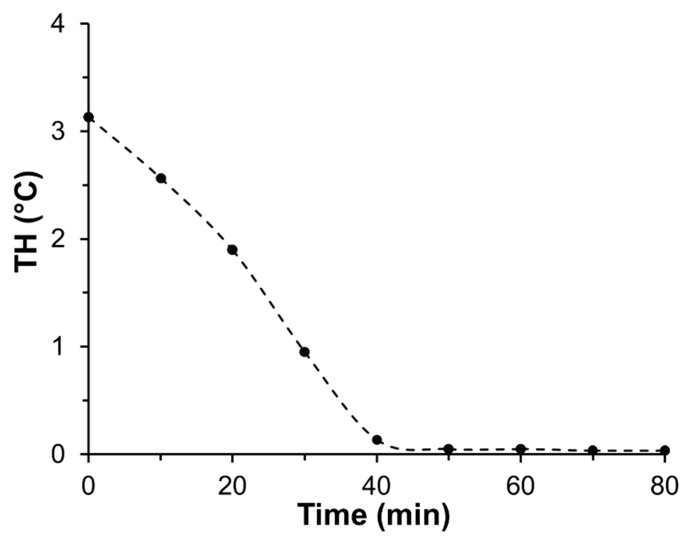
Efficiency of *Tm*AFP incorporation into a rotating ice shell. Liquid samples were drawn from the third round of *Tm*AFP purification at 10-min intervals, and TH readings were done in duplicate.

To confirm the correct amount of time over which to conduct rotary IAP, we analysed samples drawn from the liquid fraction at 10-min intervals through the 1-h process. When these were analysed for TH they showed a steady incorporation of *Tm*AFP into the ice that was complete at ~50 min (Figure 4). Thus, a 1-h period for most extractions is sufficient, which was confirmed by the near perfect recovery of *Tm*AFP through five consecutive rounds of rotary IAP (Table 1).

### 3.5. TmAFP Isoform Synergy

**Figure 5:**
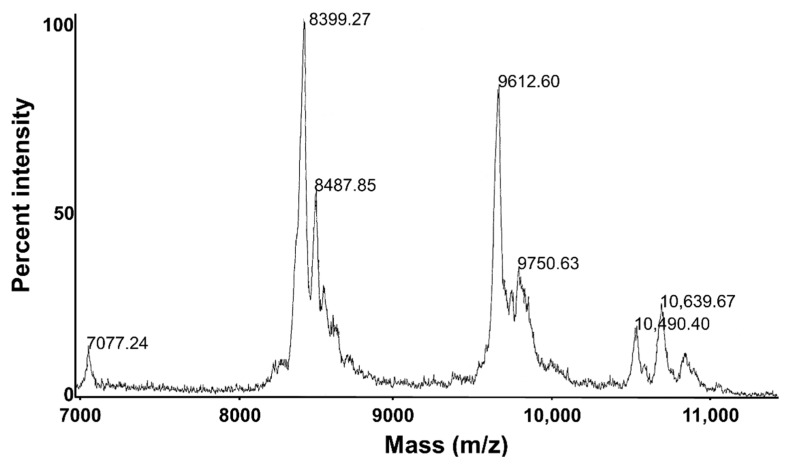
MALDI whole mass spectrometry of ice-affinity-purified native *Tm*AFP. The molecular weights (Da) of individual isoforms are indicated above the major protein peaks.

**Figure 6:**
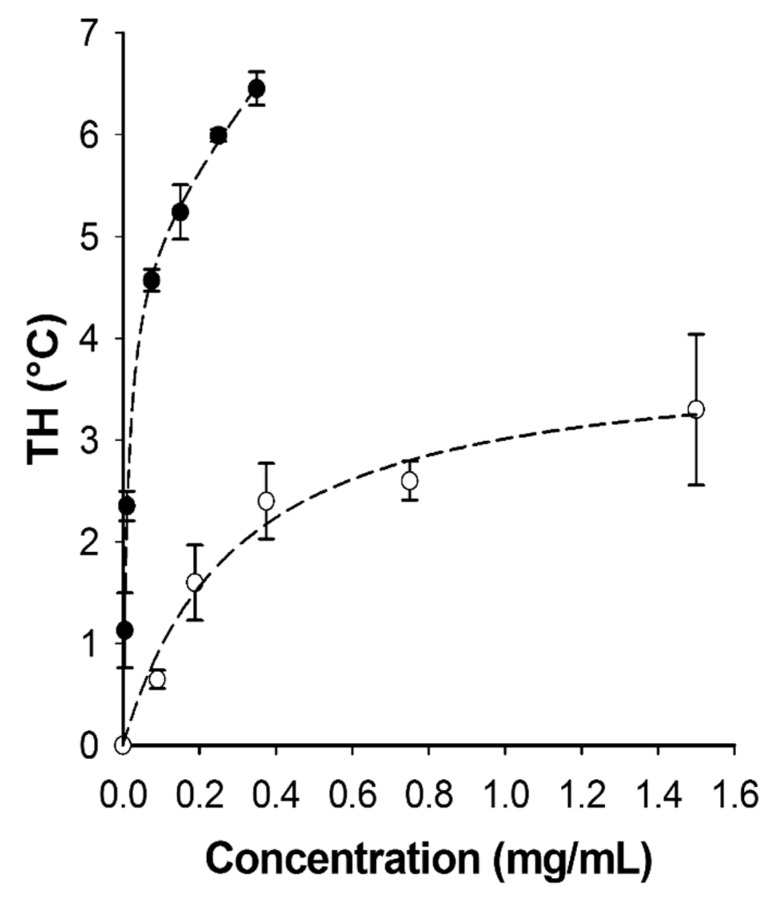
Antifreeze activity comparison of ice-affinity-purified native *Tm*AFP and a recombinantly expressed single isoform (4-9). Thermal hysteresis (TH) in °C is plotted as a function of protein concentration (mg/mL). The values for 4-9 (open circles) were taken from Marshall et al., 2002 [23]. The values for the ice-affinity purified natural *Tm*AFP isoform mixture (solid circles) were obtained in this study. TH readings were done in triplicate and error bars represent ± SD.

Matrix-assisted laser desorption/ionization (MALDI) mass spectrometry showed the purified *Tm*AFP is indeed a mixture of several isoforms (Figure 5), which was expected from previous investigations [16]. The main peaks at 7+, 8+ and 9+ kDa correspond to isoforms with 6, 7 and 8 beta-helical coils, respectively, mixed in with glycoforms within the 9+ and 10+ kDa range. The plot of TH vs. *Tm*AFP concentration (Figure 6) shows that the mixture of isoforms is several times more active than a single recombinantly prepared isoform like 4-9 [23]. For example, the mixture has 5.7 °C of TH at 0.2 mg/mL, whereas recombinant *Tm*AFP isoform 4-9 had a TH activity of 1.7 °C at this concentration. 

### 3.6. Toxicity Testing

**Figure 7:**
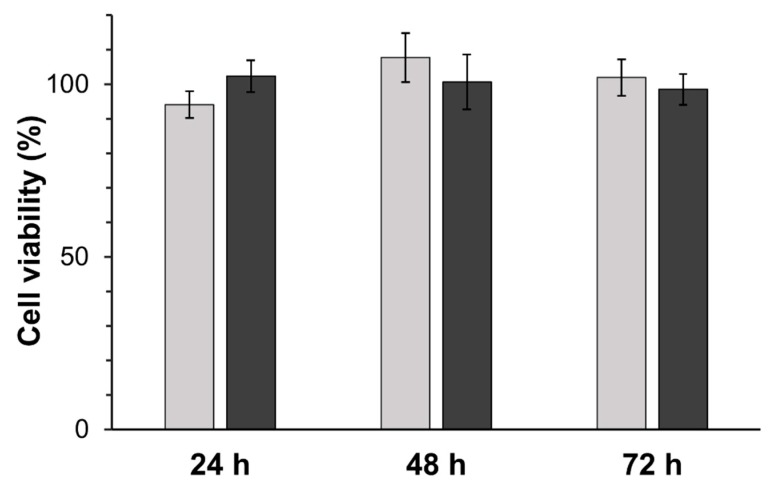
Cell viability of HEK 293 T cells after 24-, 48-, and 72-h growth periods in the presence of two different concentrations of *Tm*AFP. Light grey signifies 50 µg/mL of *Tm*AFP, and dark grey 100 µg/mL of *Tm*AFP. Cell viability was assessed through MTT reduction, and viability is shown as a percentage of control cells. Cell viability experiments were performed using four different preparations of *Tm*AFP and error bars represent ± standard error of the mean (SEM).

*Tm*AFP prepared by five rounds of IAP is not toxic to cells in culture. This was evaluated by incubating HEK 293 T cells at two different concentrations of *Tm*AFP, followed by assessing cell viability using MTT assays. No significant difference in cell viability was observed between cells incubated in the presence of 50 µg/mL and 100 µg/mL *Tm*AFP for 24, 48, and 72 h (two-way analysis of variance (ANOVA), *p* > 0.05). Mean viabilities on both treatments after the three growth periods were 100 ± 7% compared to *Tm*AFP-free control cells (Figure 7).

### 3.7. Recycling of Used TmAFP

Following the toxicity study, active *Tm*AFP was recovered from used cell culture medium. After four rounds of ice-shell purification, the amino acid composition of the recovered *Tm*AFP was consistent with unused preparations. There were some losses as only 50% of the material originally added to the medium was recovered (data not shown). 

## 4. Discussion

We report here a highly reproducible method for rapidly producing ultra-pure natural AFP that at modest concentrations can depress the freezing point of a solution by up to 6 °C on top of any colligative freezing point depression from added solutes. Two advantages of the purification method are that it does not require expensive equipment, and it can be completed within one day. Also, because the method is based on affinity of the AFPs for ice, it enables the simultaneous purification of all isoforms naturally present in the insect, and with their posttranslational modifications in place. There is significant synergy between the isoforms that makes the mixture substantially more active than an individual isoform.

It would be difficult to reconstitute this mixture using recombinant *Tm*AFP isoforms. The most abundant 7-coil isoform has 16 cysteines that must form the correct eight disulfide bonds. This was initially achieved by slow oxidation of bacterial lysates [13]. But subsequently, folding and oxidation was more efficiently achieved inside *E. coli* by using the special Origami B cell line [14]. Duplicating *Tm*AFP’s glycosylation in bacteria is not currently possible. Moreover, production of *Tm*AFP in glycosylation competent yeast strains has led to much off-target O-linked modification, presumably due to the high Thr and Ser content (30%) [24].

There are options for scaling this natural process to industrial levels. The only ingredients needed to raise *T. molitor* beetle larvae are bran and water, and the current cost for wheat bran is less than US$100 per metric ton. Climate control and lighting needs are minimal, and are suitable for rural or industrial facilities. The IAP procedure can be scaled to a much larger vessel size with multiple units simultaneously operating. Alternatively, the falling water ice-affinity purification method that uses commercial ice making machines can be employed [25]. These procedures might be sufficient to produce food-grade *Tm*AFP, especially since *T. molitor* larvae are an accepted food source and are rich in protein [26]. 

It is not clear what requirements and restrictions might be imposed on natural ice-affinity- extracted *Tm*AFP before it is an acceptable starting material for medical applications like organ storage. However, there are many suitable polishing steps that might increase quality control of the product without losing the natural enhancement of the *Tm*AFP isoform mixtures. Size-exclusion chromatography with the collection of proteins in the narrow size window of 7 to 11 kDa could be used to remove trace amounts of larger impurities. Also, preparative reversed-phase high-performance liquid chromatography (HPLC) could be used to purify the individual *Tm*AFP isoforms, which would then be recombined in an appropriate ratio for enhancement. One big advantage of the natural protein mixture is that it does not require the extra vetting and regulatory approval associated with a recombinant protein. Also, being natural rather than recombinant might lead to greater acceptance in certain markets such as the European Union, which strictly regulates the commercialization of genetically modified products.

## 5. Conclusions

Highly-active AFPs were efficiently isolated from their native source (mealworm beetles, *T. molitor*) based only on affinity for ice. The AFP isoform mixture had superior activity relative to a single recombinantly expressed isoform. Sufficient quantities of AFP (~ 4.6 mg/100 g of larvae) were obtained to prevent freezing in tests of cell and organ preservation at temperatures down to −6 °C. 

## Figures and Tables

**Table 1:** Analysis of *Tm*AFP purification through five rounds of ice-affinity purification. Total protein measured by the Bradford assay and TH readings were performed on liquid and ice fractions from *Tm*AFP purifications. All readings were done in triplicate, and all samples were assessed on an equal volume basis (400 mL), except for ice 5 with a final volume of ~275 mL. Total protein (mg) represents total quantity of protein in each 400 mL fraction, while total protein (%) represents the total protein in each fraction as a percentage of the supernatant. ± represents standard deviation (SD). Due to *Tm*AFP’s inability to bind Coomassie blue, this protein is present but not detected (n.d.) in Ice 4 and Ice 5.

Sample	Total Protein (mg)	Total Protein (%)	TH (° C)
**Supernatant**	44,301 (± 1206)	100	3.51 ± 0.43 °C
**Liquid 1**	41,160 (± 133)	92.9	0.24 ± 0.31 °C
**Ice 1**	3889 (± 28)	8.7	3.45 ± 0.58 °C
**Liquid 2**	2208 (± 24)	4.9	0.16 ± 0.43 °C
**Ice 2**	506 (± 16)	1.1	3.22 ± 0.88 °C
**Liquid 3**	493 (± 15)	1.1	0.09 ± 0.14 °C
**Ice 3**	34 (± 1.6)	0.07	3.47 ± 0.68 °C
**Liquid 4**	29 (± 2)	0.06	0.03 ± 0.01 °C
**Ice 4**	n.d.	N/A	3.55 ± 0.36 °C
**Liquid 5**	n.d.	N/A	0.06 ± 0.03 °C
**Ice 5**	n.d.	N/A	3.73 ± 0.59 °C

**Table 2:** Percent amino acid composition of four *Tm*AFP preparations (1–4). *Tm*AFP #4a and 4b were analyses done on the same sample, but with cysteine oxidized to cysteic acid in 4b to measure its % composition. Predicted percent compositions (second column) were calculated from a mixture of the four most abundant isoforms (4-9, D-16, 1-2, A-3) in the percentage ratio of 50/25/15/10, respectively [16]. Samples #1-4a were normalized to a predicted cysteine content of 19%. Asx represents the sum of Asp and Asn, which amino acids are not distinguishable in this analysis. Similarly, Glx represents Glu + Gln. n.d. – not determined.

A.A.	Predicted (%)	*Tm*AFP #1 (%)	*Tm*AFP #2 (%)	*Tm*AFP #3 (%)	*Tm*AFP #4a (%)	*Tm*AFP #4b (%)
**Asx**	13.0	13.4	13.8	12.8	13.1	14.7
**Glx**	3.9	4.0	3.9	4.3	3.7	4.6
**Ser**	7.6	7.3	7.6	6.6	7.4	7.8
**Gly**	8.2	8.3	8.4	8.5	8.4	9.4
**His**	3.2	2.2	2.4	2.4	2.3	1.9
**Arg**	0.7	1.2	1.0	1.0	1.2	1.2
**Thr**	21.9	20.9	22.2	21.8	21.4	18.8
**Ala**	11.0	10.1	10.4	10.7	10.2	11.1
**Pro**	2.4	2.4	2.5	2.5	2.5	2.9
**Tyr**	1.2	1.5	1.3	1.6	1.5	0.5
**Val**	2.5	2.7	2.4	2.8	2.6	2.7
**Met**	0.0	0.3	0.2	0.3	0.4	0.3
**Cys**	19.0	n.d.	n.d.	n.d.	n.d.	17.2
**Ile**	0.0	0.4	0.3	0.5	0.5	0.4
**Leu**	0.0	0.8	0.4	0.9	0.8	0.8
**Phe**	1.2	1.5	1.0	1.3	1.3	1.6
**Lys**	4.3	4.1	3.0	3.0	3.9	4.1
**Total**	**100.0**	**81.0**	**81.0**	**81.0**	**81.0**	**100.00**
